# Evolution of Bacterial “Frenemies”

**DOI:** 10.1128/mBio.00675-17

**Published:** 2017-05-23

**Authors:** Sophie E. Darch, Carolyn B. Ibberson, Marvin Whiteley

**Affiliations:** Department of Molecular Biosciences, Institute for Cellular and Molecular Biology, LaMontagne Center for Infectious Disease, The University of Texas at Austin, Austin, Texas, USA

**Keywords:** *Pseudomonas aeruginosa*, *Staphylococcus aureus*, aggregates, biofilms, coinfection, cystic fibrosis, mucoidy, polymicrobial infection

## Abstract

Chronic polymicrobial infections are associated with increased virulence compared to monospecies infections. However, our understanding of microbial dynamics during polymicrobial infection is limited. A recent study by Limoli and colleagues (D. H. Limoli, G. B. Whitfield, T. Kitao, M. L. Ivey, M. R. Davis, Jr., et al., mBio 8:e00186-17, 2017, https://doi.org/10.1128/mBio.00186-17) provides insight into a mechanism that may contribute to the coexistence of *Pseudomonas aeruginosa* and *Staphylococcus aureus* in the cystic fibrosis (CF) lung. CF lung infections have frequently been used to investigate microbial interactions due to both the complex polymicrobial community and chronic nature of these infections. The hypothesis of Limoli et al. is that the conversion of *P. aeruginosa* to its mucoidy phenotype during chronic CF infection promotes coexistence by diminishing its ability to kill *S. aureus*. Highlighting a new facet of microbial interaction between two species that are traditionally thought of as competitors, this study provides a platform for studying community assembly in a relevant infection setting.

## COMMENTARY

A key question underlying investigations into microbial interactions is how can bacteria coexist in the face of competition? As a model system, chronic infection of the cystic fibrosis (CF) lung is excellent for elucidating microbial interactions that shape community composition and function. CF is an inherited condition caused by mutation of the cystic fibrosis transmembrane conductance regulator (CFTR), which results in the accumulation of mucus (sputum) in patient lungs (1). Bacteria use sputum as an energy source to grow to high densities in the CF lung, causing infections that begin in early childhood and persist throughout an individual’s life (2, 3). Despite extensive knowledge of the complex nature of CF lung infections, there is still a lack of understanding regarding how this diversity is maintained. Of particular interest is the interaction of *Pseudomonas aeruginosa* and *Staphylococcus aureus*, two of the most prominent bacteria in the CF lung (2, 3). It is well-known that *S*. *aureus* is often one of the first bacteria to infect the CF lung during childhood, but it is subsequently outcompeted and displaced by *P*. *aeruginosa*, which produces an arsenal of quorum sensing (QS)-regulated virulence factors that are lytic to *S*. *aureus* (4). However, the mechanisms that allow *S*. *aureus* and *P*. *aeruginosa* to coexist during CF lung infection are not well understood.

In a recent article, Limoli and colleagues provide evidence that the conversion of *P*. *aeruginosa* to mucoidy during chronic CF infection promotes coexistence with *S*. *aureus* by dampening the ability of *P*. *aeruginosa* to kill *S*. *aureus* (5). Specifically, they found that in standard *in vitro* culture conditions, survival of *S*. *aureus* was limited when it was cultured with wild-type *P*. *aeruginosa*. However, when cultured with *P*. *aeruginosa* strains that had been isolated alongside *S*. *aureus* from CF sputum samples, *S*. *aureus* survival improved. Further examination of these isolates showed that many had genetic mutations that led to the overproduction of alginate. The model of mucoid conversion proposed by Limoli et al. is strongly supported by the data presented, despite the fact that mutation as a primary requirement for *P*. *aeruginosa* coexistence with *S*. *aureus* is not a well-supported idea in the existing literature. While the conversion of *P*. *aeruginosa* to a mucoid phenotype in the lung has long been correlated with a poorer clinical outcome (6, 7), populations of *P*. *aeruginosa* isolated from CF lungs have been shown to be highly diverse (8), with a mixture of mucoid and nonmucoid phenotypes, despite many months of chronic infection (9). In addition, alternative mutations such as those involved in QS-regulated lytic pathways are frequently observed. Therefore, it is likely that many of these mutations are a result of selective pressures that allow *P*. *aeruginosa* to persist within the host, not directly as a result of coexistence with another microbe, despite their impact on microbial community interactions.

**FIG 1:**
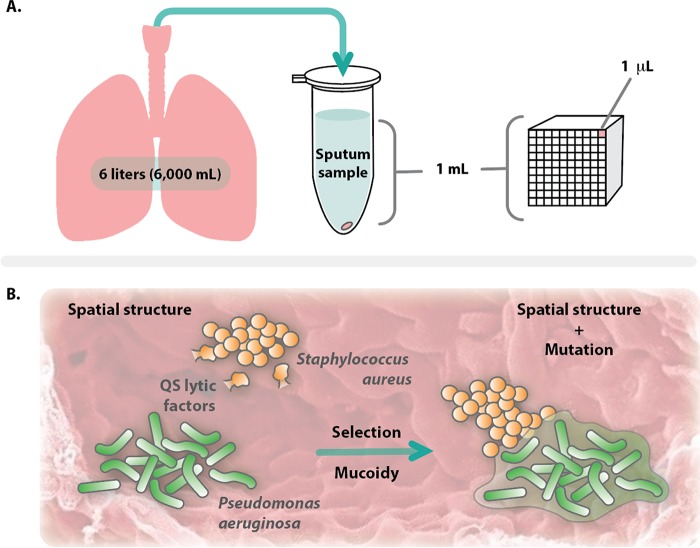
Scale and spatial structure impact interactions between microbes in the CF lung. (A) Scale comparison of bacteria residing in the CF lung. The structure of the CF lung provides both a large surface area and volume. From an expectorated 1 ml sputum sample, approximately 10^9^ bacteria occupy ~1 μl of this volume. The observation that bacteria exist as aggregates in the CF lung that must be localized within microns in order to interact suggests either that aggregates are either geographically isolated or in concentrated sites. (B) When aggregates are in concentrated sites, coexistence of *S. aureus* and *P. aeruginosa* is likely maintained by spatial structure, preventing lysis of *S. aureus* by QS-regulated lytic factors produced by *P. aeruginosa*. Selective pressure applied by the host often causes mucoid conversion of *P. aeruginosa*, which in turn alters spatial structure and changes how these two bacteria coexist.

At the macroscale, the CF lung provides a vast landscape in which a bacterial community can develop. A single CF sputum sample often contains ~10^9^ bacteria per ml; however, a bacterial population of this size would volumetrically fill less than 0.1% of 1 ml of sputum (Fig. 1A). In addition, there is evidence that bacteria infecting the CF lung exist as small, high-density clusters of cells (~10^1^ to 10^4^ cells), called aggregates, that form structured and spatially organized communities (10–13). Thus, it may be valuable to consider the CF lung as a vast landscape occupied by sparse bacterial aggregates. Previous studies indicate that aggregates need to be within microns of each other to interact (14, 15), and therefore, it is valuable to consider interactions on the micron scale, rather than as a macroscale clinical sample taken from the lung.

It is well-known that when grown in well-mixed communities *in vitro*, *P*. *aeruginosa* actively kills *S*. *aureus*, a finding supported by Limoli et al. (5). However, there is evidence that *P*. *aeruginosa* and *S*. *aureus* can coexist for days in the absence of mutation when grown in spatially structured communities. For example, the Lubbock wound model promotes coexistence of *P*. *aeruginosa* and *S*. *aureus* for multiple days, even with *P*. *aeruginosa* strains that are highly lytic for *S*. *aureus* in well-mixed environments (16, 17). The coexistence of these bacteria has been shown to result in their increased tolerance to antimicrobial agents, both in human and murine models of infection, indicating that there can be fitness benefits for both species as a result of their interaction (17, 18). Additionally, work by Fazli et al. has shown that *P*. *aeruginosa* and *S*. *aureus* occupy distinct spaces during human infection, highlighting a role for spatial structure (19). It would be surprising if biogeography did not play a role in the interaction described here. Similar to alginate, there is increasing support that large polymers such as mucin, DNA, and poly-*N*-acetylglucosamine play a role in spatially structuring microbial communities during infection (15, 20, 21). For example, our lab has demonstrated that *Aggregatibacter actinomycetemcomitans* and *Streptococcus gordonii* interact through cross-feeding, and that this interaction is maintained by precise spatial organization of these organisms through modulation of poly-*N*-acetylglucosamine levels in the biofilm matrix (15). Additionally, the secreted biofilm matrix of *Candida albicans* was found to be protective for *S*. *aureus* against antimicrobial treatment (22). Such polymers likely play multiple roles in these systems, acting as both a physical barrier and a chemical sink to small molecules. Therefore, an alternate hypothesis for the findings by Limoli et al. (5) is that the alginate produced is not only reducing the production of lytic factors by *P*. *aeruginosa*, but may also promote spatial structuring that allows for coexistence (Fig. 1B).

A great strength of this study is the effective use of an *in vitro* epithelial cell model that supports the coexistence of *P*. *aeruginosa* and *S*. *aureus*, pushing the biological relevance of this observed interaction beyond well-mixed *in vitro* batch culture methods that do not provide realistic environments in which to study microbe-microbe interactions (12, 23). The use of the epithelial model in this study raises interesting questions about the spatial structure of the developing *P*. *aeruginosa*-*S*. *aureus* community. Do the bacteria form aggregates in this system? Is there spatial patterning? Limoli and colleagues (5) have provided an experimentally elegant approach for observing how the host drives selection of phenotypes that potentially alter the ways in which bacteria interact *in vivo*. Understanding more about these interactions at the micron scale will inform us on how these communities assemble and potentially how to disrupt them. This study provides a platform for some exciting future studies to elucidate the coexistence of two pathogens relevant not only to the CF lung environment, but also to other infection sites such as chronic wounds.
